# Older Australians Can Achieve High Adherence to the Mediterranean Diet during a 6 Month Randomised Intervention; Results from the Medley Study

**DOI:** 10.3390/nu9060534

**Published:** 2017-05-24

**Authors:** Courtney Davis, Jonathan Hodgson, Janet Bryan, Manohar Garg, Richard Woodman, Karen Murphy

**Affiliations:** 1 Alliance for Research in Exercise, Nutrition and Activity, School of Health Sciences, University of South Australia, GPO Box 2471, Adelaide, SA 5001, Australia; karen.murphy@mymail.unisa.edu.au; 2 School of Medical and Health Sciences, Edith Cowan University, 270 Joondalup Dr., Joondalup, WA 6027, Australia; jonathan.hodgson@ecu.edu.au; 3 School of Medicine and Pharmacology, University of Western Australia, 35 Stirling Highway, Perth, WA 6000, Australia; 4 School of Psychology, Social Work and Social Policy, University of South Australian, GPO Box 2471, Adelaide, SA 5001, Australia; janet.bryan@unisa.edu.au; 5 Nutraceuticals Research Program, School of Biomedical Sciences and Pharmacy, University of Newcastle, Callaghan, NSW 2308, Australia; manohar.garg@newcastle.edu.au; 6 Flinders Centre for Epidemiology and Biostatistics, Flinders University, GPO Box 2100, Adelaide, SA 5001, Australia; richard.woodman@flinders.edu.au

**Keywords:** Mediterranean diet, adherence, Australia, nutrients, food intake, elderly

## Abstract

Adherence to a Mediterranean diet (MedDiet) is thought to be achievable in non-Mediterranean regions, but this has yet to be investigated. We aimed to determine if an older Australian population could adhere to a MedDiet for six months. We conducted a randomised, parallel dietary intervention trial with two dietary arms: the Mediterranean diet (MedDiet) group and the habitual diet (HabDiet) control group. A 15-point Mediterranean diet adherence score and food and nutrient intakes were estimated from three-day weighed food records collected at baseline, two and four months. Erythrocyte fatty acids, serum carotenoids and urinary metabolites were assessed at baseline, three and six months. We enrolled 166 participants; 152 commenced and 137 completed the study (70 in the MedDiet group, 67 in the HabDiet group). Adherence scores were significantly higher in the MedDiet group at two months (between group difference 2.2, 95% CI 1.3, 2.9) and four months (between group difference 2.6, 95% CI 1.9, 3.3). Consumption of vegetables, fruits, fish, legumes, nuts and olive oil significantly increased in the MedDiet group compared to the control, and discretionary food intake decreased (*p* < 0.01). Measures of compliance including serum β-carotene, lycopene and erythrocyte monounsaturated fatty acids were significantly higher in the MedDiet group at three and six months (*p* < 0.05). Our results indicate that a population of older Australians can adopt a Mediterranean diet over a six month period.

## 1. Introduction

In the mid-20th century, evidence emerged that populations living in the Mediterranean region had lower incidence of cardiovascular disease (CVD) than those living in northern Europe and North America [1]. It was suggested that the distinguishing feature between these regions was diet, particularly the fat composition [2]. The Greek population was consuming relatively little processed foods, red meat and saturated fat, but consumed a larger amount of plant foods including olive oil as the main culinary fat, providing monounsaturated fatty acids (MUFA), micronutrients, fibre and antioxidants [3]. This dietary pattern was termed the Mediterranean diet (MedDiet) [4].

The MedDiet has received much attention from researchers with the majority of studies reporting positive health outcomes when close adherence is achieved [5,6]. Large observational studies including the European Prospective Investigation into Cancer and Nutrition (EPIC), Finland, Italy, the Netherlands Elderly (FINE), and Healthy Ageing: a Longitudinal study in Europe (HALE) studies, the Survey in Europe on Nutrition and the Elderly (SENECA) study, and the Nurses’ Health Study have been meta-analysed by Sofi et al., most recently in 2014 [6,7,8,9,10,11]. With a database of over four million subjects, their results showed overall mortality risk was reduced by up to 32% when comparing lowest with highest MedDiet adherence level [6]. For every two-point increase in adherence score (maximum 9), the relative risk for CVD mortality/incidence was 0.90 (95% CI 0.87–0.92). The landmark Prevención con Dieta Mediterránea (PREDIMED) study is the largest Mediterranean diet randomised intervention trial completed to date, involving ~7500 Spanish adults with a median follow-up close to five years [12]. The primary outcome, risk of CVD (stroke, myocardial infarction or angina), was reduced by 30% after following the MedDiet, and risk of incident diabetes was reduced by 52% compared with a low fat control diet [13]. 

This evidence led the US Department of Health and Human Services and US Department of Agriculture to recognise and include the MedDiet as one of two alternative healthy dietary patterns in the 2015–2020 US Dietary Guidelines [14]. The US population, along with other Western nations including Australia and the UK has sub-optimal intakes of fruits and vegetables but higher intakes of discretionary foods than Mediterranean populations. These countries also suffer from a high prevalence of obesity, diabetes and CVD. In Australia, discretionary foods contribute over 35% of energy intake and only 4% of the population consumes the recommended servings of vegetables [15]. Cardiovascular disease is the leading cause of death, responsible for 30% of mortality cases in 2012 [16]. Furthermore, with populations ageing in Western nations, rates of chronic disease are likely to increase, placing economic burden on health care systems [17]. Age is a leading risk factor of CVD and dementia, and so intervening with strategies to reduce the burden of these diseases is critical at the present time. 

Epidemiological evidence shows that non-Mediterranean populations with a priori measured high adherence to a MedDiet have lower risk of CVD [11,18]. Indeed, in the meta-analysis by Sofi et al., of 23 studies added to the 2014 analysis, 11 were from non-Mediterranean populations, nine were from Mediterranean populations and three were multicentre studies with both Mediterranean and non-Mediterranean countries. The MedDiet may offer a dietary strategy to reduce morbidity and mortality from chronic disease in non-Mediterranean countries. It has been suggested that the adaptability and variety of the diet makes it transposable to non-Mediterranean countries [18,19,20]. The limitation of the meta-analysis is the inclusion of observational studies only. There are few longer term intervention trials to assess how successfully non-Mediterranean populations can change their diets. Consequently, it remains unknown whether it is plausible for regions beyond the Mediterranean Sea to adopt the MedDiet. Barriers may include cultural beliefs, palatability, food access, cost, time for food preparation/shopping, and environment (for example proximity to fast food restaurants and access to discretionary foods) [21]. Whether the Australian population can adopt the MedDiet ad libitum over the longer term is unknown. We conducted a randomised controlled intervention trial in an older Australian population to determine how well this population could adopt the MedDiet in 6 months. The primary outcome for this study is adherence to the MedDiet measured via a 15-point score adapted from Trichopoulou et al. [22]. Secondary outcomes include self-reported food, nutrient, carotenoid and flavonoid intake from weighed food records and biomarkers of adherence. In addition, qualitative data are presented highlighting participant experiences with consuming the MedDiet. 

## 2. Materials and Methods 

The Mediterranean diet for cardiovascular and cognitive health in the elderly study (MedLey) protocol has been published [23,24], and will be summarised. The study was a 6-month randomised controlled clinical dietary intervention trial. The primary aim was to assess the effect of the MedDiet on cognitive function after 6 months compared to the habitual Australian diet. Secondary aims included cardiovascular risk factors and the ability of Australians to adhere to the MedDiet. The results of the latter are presented here. Participants were recruited from metropolitan Adelaide between July 2013 and May 2014. Local television and newspapers (Messenger/Advertiser) were the primary methods of advertisement for the study. Interested volunteers left their details with the clinic, and were then called to confirm interest and sent a screening questionnaire via post or email. If eligible based on this questionnaire, volunteers attended a screening visit where blood samples, blood pressure, height, weight, waist circumference and cognitive function via DemTect were assessed to determine eligibility. Exclusion criteria included the following: persons considered by the investigator to be unwilling, unlikely or unable to comprehend or comply with the study protocol, age < 65 years, previous/current traumatic head/brain injury, neurological or psychiatric conditions, previous stroke, unstable use of anti–depressant medication (<6 months), use of medication to treat anxiety, current or recent (in the last 6 months) malignancy, major liver, kidney, respiratory or gastrointestinal disease, current cardiovascular disease or angina, uncontrolled hypertension (>170/100), current smoker, vegetarian (does not eat red meat, poultry or fish), actively undertaking weight loss program, use of appetite suppressants or Orlistat (Xenical), age and education adjusted scores of <13 on the DemTect, or body mass >135 kg (limit on dual energy X-ray absorptiometry (DEXA) scanner). Participants were stratified by age, gender and BMI and randomised into either the MedDiet group or the habitual diet (HabDiet) group by the process of minimisation. A chief investigator not involved in any participant contact performed the randomisation. Participants were listed by studyID, age, gender and BMI in an excel spreadsheet which was sent to the chief investigator. They then placed each ID into one of the two groups at random, so that age, BMI and gender averages remained similar between groups. We randomised 166 participants (*n* = 85 MedDiet group, *n* = 81 HabDiet group), and 14 withdrew before commencement leaving 152 (*n* = 80 MedDiet group, *n* = 72 control group) who commenced the study. Ten withdrew from the MedDiet group and 5 from the HabDiet group, thus 137 completed their 6 months visit (11% attrition from commencement). The study was conducted in 2 cohorts, the first (*n* = 69) conducted between August 2013 and April 2014, the second (*n* = 83) between May 2014 and February 2015. Those in the MedDiet group were prescribed a MedDiet for 6 months (energy balanced for individual requirements), while those in the HabDiet group followed their regular diets for 6 months in a parallel design. We aimed to assess dietary adherence through self-reported 3-day weighed food records (WFRs), daily checklists and also objective measures: fasting serum carotenoid levels, erythrocyte fatty acids and 24-h urinary metabolites. Dietary intake was assessed at baseline, 2 and 4 months. Urinary metabolites, erythrocyte fatty acids and serum carotenoids were assessed at baseline, 3 and 6 months. All clinic visits occurred at the Sansom Institute for Health Research, University of South Australia. Checklists were completed daily by those randomised to the MedDiet group only. Further to this, in an exit survey participants were asked to report on their experiences in relation to following the MedDiet. The study was conducted according to the guidelines laid down in the Declaration of Helsinki and all procedures involving human subjects/patients were approved by the University of South Australia Ethics Committee (#31163). Written informed consent was obtained from all subjects/patients with an investigator present to answer and clarify any queries. Participants received $100 pro rata as an honorarium. The trial was registered with the Australia New Zealand Clinical Trials Register (ACTRN12613000602729), on the 27 May 2013 [25].

### 2.1. The Mediterranean Diet

The diet was based on a literature review to determine approximate food and nutrient content of the MedDiet [26] and was tested in a pilot study in a population group similar to that of the MedLey study [27]. The review included descriptive studies, observational and intervention diets, including data from the EPIC and PREDIMED studies [12,28]. A recommendation for daily and weekly servings of key foods and the resulting nutrient content was determined. Based on their energy requirements and in line with a traditional MedDiet, volunteers were asked to consume the following: 1–3 tablespoons of extra virgin olive oil, 5–6 servings of vegetables, 2–3 servings of fruit, 4–6 servings of grain foods, up to 1 white potato, up to 200 mL of red wine and up to 1 cup of skim milk per day, and 4–6 servings of nuts, 6 servings of Greek yoghurt, 3–4 servings of cheese, 1–3 servings of poultry/pork, 3 servings of fish, 3 servings of legumes, 1–2 servings of small goods, up to 1 serve of red meat, up to 6 eggs, and up to 3 servings of discretionary foods per week. To adjust for differences in estimated energy requirements, recommended servings of key foods was reduced to create 3 additional lower energy diets, which nonetheless retained a nutrient profile in line with MedDiet principles. The nutrient content of the four energy levels is published elsewhere (Davis et al. [23]). 

### 2.2. Intervention Strategy 

Participants were educated at baseline by a study dietitian. They received written resources detailing the basic principles of the MedDiet, a list of Mediterranean fruits and vegetables, a list of serving sizes for key foods, recommendations for number of servings to consume daily and weekly, suggestions for recipe modification and eating out, a recipe book with appropriate Mediterranean recipes and checklists to record their daily intake of servings. They were encouraged to eat a variety of foods, have cooked and raw vegetables, use olive oil in baking, cooking and as a dressing/sauce and consume 50% of their cereals from whole-grain sources. This education session lasted between 45 min to 1 h. Participants were provided with foods to aid adherence, including extra virgin olive oil (750 mL per fortnight), plain and flavoured low fat Greek yoghurt (1 kg per week), unsalted peanuts, almonds and walnuts (~350 g per week), canned legumes and canned tuna. Combined, this was 30–35% of estimated energy requirements. These foods were chosen because they were reasonably shelf-stable, somewhat expensive but important components of the diet, and were offered every 2 weeks. After the initial baseline session, participants attended a 30 min session with the dietitian biweekly to discuss the diet, return checklists, have body mass assessed and collect food. At these sessions, advice tailored to each individual was given to help participants adhere closely to the diet. We aimed to maintain body mass to avoid the possible confounding effects of weight loss on outcomes, such as lipids and blood pressure. If weight loss or gain was occurring, personalised advice was given to counteract this, by either increasing key foods such as olive oil, whole-grain cereals, fruits and vegetables, or decreasing certain foods such as discretionary foods. In addition, participants could call or email the dietitian at any time during the week with questions or concerns. Other than these supports, participants were required to adapt to the MedDiet in their own capacity. 

### 2.3. Three-Day Weighed Food Records

Weighed food records were undertaken by participants in the week preceding commencement of the intervention to assess baseline dietary intake. They were then repeated between weeks 9–11 (2 months) and weeks 19–21 (4 months) during the intervention phase. WFRs were administered at the biweekly appointments rather than 3 and 6 month clinic visits to reduce participant burden. Participants were asked to record all food and beverage consumption on consecutive week days and one weekend day, i.e., either Thursday, Friday and Saturday, or Sunday, Monday and Tuesday. All participants received detailed verbal and written instructions from trained staff, digital kitchen scales and a paper diary in which to write their food intake. Upon return these were checked for completeness with the participant. Recorded foods were analysed using FoodWorks Professional Version 7.0.3016 (Xyris Software Spring Hill, QLD, Australia), by a study dietitian and exported to Microsoft Access™ (Microsoft Corporation, Redmond, WA, USA) for analysis. Total quantity of flavonoids and carotenoids was calculated for all available foods from the following databases: United States Department of Agriculture (USDA) Database for the Flavonoid Content of Selected Foods (Release 3.1 and 2.1), USDA-NCC Carotenoid Database for U.S. Foods, and the USDA Database for the Isoflavone Content of Selected Foods (Release 2.0). Where necessary, data were supplemented with information from the online phenol explorer. To assess adherence to the MedDiet, we used a 15-point scoring system modified from the 9-point MedDiet score (MDS) created by Trichopoulou et al. [22]. Total food intake as grams/MJ was divided into 15 predefined food groups. Using the baseline group specific means as cut-offs, each participant scored either 1 point or 0 points for each of the 15 food groups: 1 point was awarded for intakes above the mean for vegetables, fruits, nuts, legumes, fish, breads, cereals, olive oil, and 1 point for intakes below the mean for sugars, eggs, dairy, potato, meat and miscellaneous foods. For red wine, intakes between 0–200 mL/day received 1 point, while above 200 mL received 0 points. Maximum score (15) reflects highest adherence. Miscellaneous foods included non-Mediterranean food items such as muesli bars, soy products, protein bars and other special dietary products, tropical fruit, and discretionary foods including confectionary, chocolate, potato crisps, deep-fried foods, alcoholic beverages other than red wine, biscuits, cakes, muffins, cupcakes, low fibre breakfast cereals, cream, butter, margarine (other than olive-oil based margarine), ice cream, bakery products and milkshakes. Beverages including tea, coffee, water, soft drinks, fruit and vegetable juices, cordials and flavoured milks were not included in the score. The adjustments to the score were for the following reasons: evidence suggests foods such as nuts and sugars may be independently associated with health outcomes, thus we separated these groups to allow future sensitivity analysis; a large amount of foods come from non-core food groups in the modern Australian diet, thus we deemed it appropriate to include a “miscellaneous” food group; we used the mean rather than median after excluding outliers, because in some cases the median intake for key food groups such as legumes was 0, which did not reflect a traditional MedDiet; we did not want vegetable intake to include white flesh potato as this can displace other vegetables in the diet; and finally instead of using gender-specific values, we controlled for differences in energy requirements and intake by using g/MJ. We did not include beverages in the final score as they were not originally included, and other than tea, water, coffee and to a lesser extent juice, intakes of other beverages were minimal. However, we did record beverage intake as a 16th group to potentially investigate further in future. 

### 2.4. Weekly Checklists 

To monitor adherence throughout the duration of the trial, all participants randomised to the MedDiet group were asked to complete a semi-quantitative checklist. On a daily basis, participants were asked to record when foods were consumed, as half or whole servings using a checklist. Discretionary foods, small goods, red meat, eggs, red wine, skim milk and white potatoes were optional foods and had no minimum requirement. Total weekly servings for each food group were summed, and a percentage adherence for each food group was calculated by comparing actual consumption with recommended consumption. For foods where there was a minimum requirement, the equation was as follows: a÷r×100 where a is the number of actual servings consumed and r is the number of recommended servings. For foods with a maximum requirement, the inverse equation was used: r ÷a ×100. If the per cent adherence was greater than 100%, it was rounded down to 100%. Total weekly adherence was calculated by averaging the adherence for each food group, and overall adherence calculated by averaging the weekly adherence percentages. Thus all food groups were weighted equally in considering per cent adherence. 

### 2.5. Serum Carotenoids, Erythrocyte Fatty Acids and 24-h Urinary Metabolites

Participants gave 8-h fasting blood samples for the analysis of erythrocyte fatty acids and serum carotenoids. Samples were taken via venepuncture, centrifuged and stored at −80 °C until analysis. Erythrocyte fatty acid composition was determined as an indicator of longer term fatty acid status using direct transesterification as described elsewhere [29]. Carotenoids were measured as an indication of fruit and vegetable consumption, from serum according to the method of Barua et al. [30] using high performance liquid chromatography. Participants collected a 24 h urine sample for analysis of excretion of key minerals magnesium, calcium, potassium and sodium at a NATA accredited laboratory. The 24 h collection period began with the second void of the day before, and continued until the first void on the day of the baseline, 3 and 6 months clinic appointment. 

### 2.6. Exit Survey

The MedLey exit survey questioned volunteers on their experiences within the trial, including aspects of following the intervention diet. Box 1 shows the three questions related specifically to the diet from the exit survey. Surveys were administered between the final two clinic appointments, which were spaced 1 week apart (i.e., during the final week of the trial). Between cohort 1 and cohort 2, two questions from the survey were removed, thus in cohort 1 the third question was number 15, and in cohort 2 it was 13. Responses to the exit survey were coded numerically for analysis. For question 9, the four possible answers were numbered 1–4 (1 = yes all of the time) and the count for each response was calculated for each food. For question 10, written answers were recorded, and based on the answers 11 categories were developed to capture the various responses. Each participants response was then grouped into one of the 11 categories: vegetables (1); attitudes of friends/family (2); restriction other specific foods or beverages (other than red meat or extras) (3); changing dietary habits (4); eating out/at friends/holidays/special occasions (5); nothing (6); olive oil (7); red meat restrictions (8); volume of food (9); white wine/beer restrictions (10); or extras restrictions (11). All answers given were coded in this way, thus some participants had multiple codes applied. For question 13/15, answers were coded as follows: 1 = all components, 2 = most components (at least 5 different aspects listed or “most components” indicated in the answer), 3 = 3–4 components listed, 4 = 1–2 components listed. The count for each code was then calculated.

Box 1 **Exit survey questions pertaining to dietary adherence.**

Question 9. The table below asks you about the foods you were asked to eat and restrict. Please tick the appropriate box:
Did you…Yes all of the timeYes most of the timeYes some of the timeNone of the time(1) Enjoy the yoghurt?



(2) Enjoy the legumes?



(3) Enjoy the tuna?



(4) Enjoy the olive oil?



(5) Enjoy the nuts?



(6) Manage the red meat restrictions?



(7) Manage the “extras” restrictions?



(8) Manage with the milk restrictions?

Question 10. What was/were the most difficult thing(s) about following the Australianised MedDiet?

Question 13/15. Which, if any, components of the MedDiet are you likely to continue with now that the study is finished?

### 2.7. Statistics

Sample size for this study was based on the primary outcome measure of cognitive function and is explained in detail elsewhere [23]. We calculated that 128 subjects (two groups of *n* = 64) would provide 80% power to detect a significant (*p* < 0.05) 0.5 standard deviation change in cognitive outcomes. For this study, adherence score was the primary outcome. With a sample size of *n* = 128, based on results from our pilot study [27] we estimated we would have >80% power to detect a significant (*p* < 0.05) 1 unit difference in the 15-point adherence score between groups (standard deviation = 2). We expected an effect size of at least a 2-unit change. Raw data is available as online supplementary material (Table S1). Continuous variables are presented as mean ± standard deviation (SD), categorical variables as count (%). Residuals were checked for normality and any non-normal variables were log10 transformed before analysis. For dietary variables including nutrient and food intakes, outliers were identified from histograms and quantile-quantile plots. If these were >3 SD from the mean and affected the mean value for diet group and visit, they were removed. In this way extreme values for energy and other nutrients were identified and removed. To compare completers and non-completers for dietary, clinical and demographic characteristics at baseline, independent samples *t*-tests were used for normally distributed variables, and Mann-Whitney *U* test for non-normally distributed variables. To assess differences in adherence scores, nutrient and food intake, urinary metabolites, erythrocyte fatty acids and serum carotenoids, analyses were performed using intention-to-treat linear mixed effects models with a group × time interaction term to determine overall differences in effects across time and at each time point. Significant was set at 0.05 (two sided). For food intakes, gender was controlled for in the model. To control for energy intakes, all food and nutrients intakes were analysed per MJ of energy intake. In a sensitivity analysis, the 10-point MDS was applied to our data, to enable comparison to previous studies. Gender-specific median intakes at baseline were used as cut-offs for baseline, 2 month and 4 month intakes. One point was awarded if intakes of vegetables, legumes, fruits and nuts, cereals, fish and monounsaturated fatty acid:saturated fatty acid (MUFA:SFA) was above the median, and 1 point awarded if intakes of meat/poultry and dairy were below the median. Additionally, 1 point was awarded for intakes between 10–50 g ethanol for males, and 5–25 g for females, so that minimum score was 0 and maximum was 9. To calculate adherence to the a priori MedDiet, we also calculated the MDS using cut-offs established from the Greek cohort enrolled in the EPIC study [22]. Linear mixed effects models were used to calculate between group differences for adherence score using these methods. The cut-offs for all three scoring methods are provided as online supplementary material (Table S2). Statistics were performed using IBM SPSS Statistics (version 21) and STATA (Version 13.0, StataCorp, College Station, TX, USA). 

## 3. Results

The first participants commenced in August 2013 and all had completed the 6-month period by February 2015. Of 152 participants who commenced, baseline data were complete for 150 participants for dietary intake and urinary metabolites, and 152 participants for erythrocyte fatty acids and carotenoids. All participants with baseline data were included in the analyses. Figure S1 (supplementary material) shows the CONSORT flow diagram for the MedLey study. One hundred thirty-seven completed the study (MedDiet = 70, HabDiet = 67). Groups were similar for age (71.0 ± 4.9 years in the MedDiet group, 70.9 ± 4.9 year in the HabDiet group) and BMI (26.7 ± 3.7 kg/m^2^ in the MedDiet group, 27.1 ± 4.1 kg/m^2^ in the HabDiet group) at baseline. Supplementary file 4 shows the CONSORT flow diagram including reasons for withdrawals. There were no significant differences between those who completed and those who withdrew for clinical and demographic characteristics, except that insulin was higher amongst withdrawals in the HabDiet group. For dietary intakes, there were no differences between completers and non-completers at baseline. Fifty eight per cent were female in the MedDiet group, 54% in the control group. Baseline demographic information has been published elsewhere [31]. Table 1 shows the baseline characteristics for carotenoids, erythrocyte fatty acids and urinary metabolites. 

### 3.1. A Priori Adherence Score and Checklists

**Figure 1:**
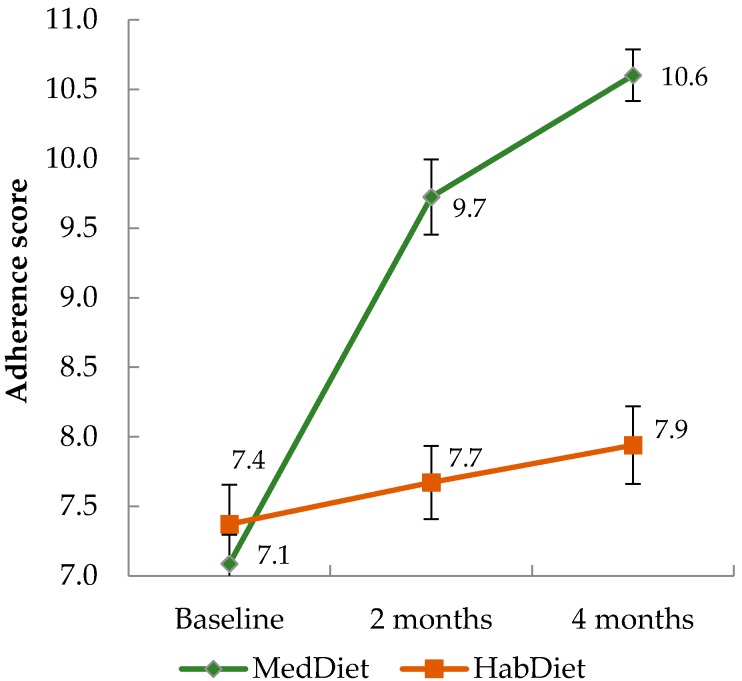
Mean adherence score at each time point by diet group. Mean (error bars depict SEM) MedDiet adherence scores of Australian men and women (age ≥ 65) at baseline, and two and four months after following a MedDiet (*n* = 80) or their HabDiet (*n* = 70). MedDiet adherence score based on intakes of 15 food groups calculated from 3-d WFRs, range 0–15 where 15 represents highest possible adherence level. Mean scores at each time point presented. Linear mixed effects model with score as the dependent variable, diet and visit as factors, and unstructured covariance used to compare groups. Analyses were intention to treat. MedDiet, Mediterranean diet; HabDiet, habitual diet; WFR, weighed food record.

Adherence scores increased in the MedDiet group significantly (*p* < 0.01), while they remained unchanged in the HabDiet group (Figure 1). Scores were significantly different between groups at two months (2.1, 95% CI 1.3, 2.9) and four months (2.6, 95% CI 1.9, 3.3), with a significant overall effect of diet on adherence (*p* < 0.001). The sensitivity analysis revealed similar changes when the 10-point MDS was applied using sex-specific baseline medians, although when the sex-specific medians from the Greek cohort of the EPIC study were used, adherence scores were lower (Table 2). According to the checklists, a mean of 91% of recommended servings were consumed, across all days and an adherence percentage of over 90% was achieved for milk, small goods, poultry, cheese, fruit, olive oil, fish, yoghurt and nuts. 

### 3.2. Food and Nutrient Intake 

**Figure 2:**
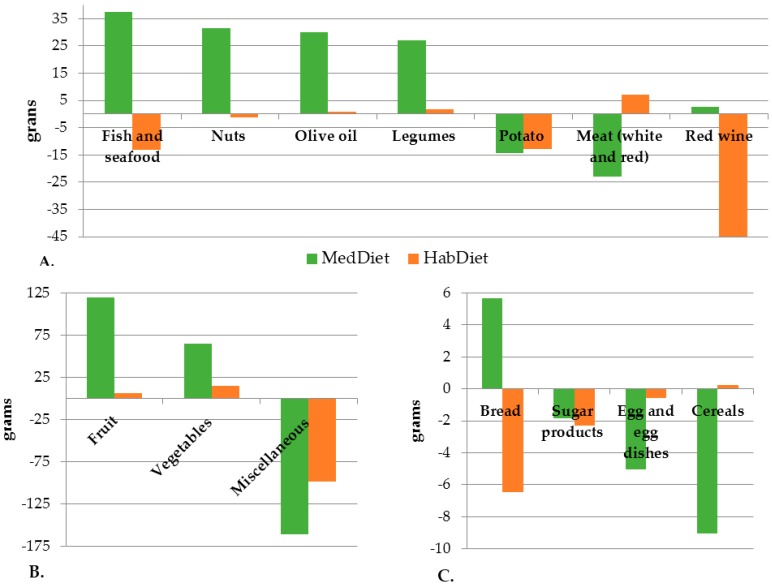
Changes in food intakes from baseline to four months, by four diet group. MedDiet (*n* = 80), HabDiet (*n* = 70): (**A**) changes between 10 and 50 g; (**B**) changes >50 g/day; and (**C**) changes <10 g per day. All changes based on the MedDiet group, with the exception of red wine for which the change was based on the HabDiet group. Miscellaneous foods included small goods, cakes, muffins, high fat crackers, pastries, sweet biscuits, high-sugar breakfast cereals, muesli bars, take away foods, chips, chocolate, confectionary <50% sugar, ice cream, beer, port, spirits, white wine. Sugar products included all foods comprising >50% sugars. MedDiet, Mediterraenan diet; HabDiet, Habitual diet.

Figure 2 shows the mean change in intake for 15 food groups. Based on Australian standard serving sizes [32], those in the MedDiet group increased their fruit, nut and vegetable intakes by approximately 1 serve per day and their dairy intake by ½ serve per day. Fish and legume consumption increased by 3 and 2.5 servings per week, respectively. Total meat consumption decreased by 25 g/day, approximately 0.25–0.3 servings. The largest change amongst both groups was a significant decrease in miscellaneous foods. Changes amongst the HabDiet group were otherwise minimal. Table S3 (supplementary material) shows the nutrient intakes, by diet group and gender at baseline and six months, compared to Australian guidelines [32]. 

Table 3 shows the changes in nutrient intake between baseline and two and four months within and between groups. Table 4 highlights these changes for food intakes. Within the MedDiet group, energy intakes did not change, nor did the contribution of protein or alcohol to total energy, vitamin C, folate, vitamin A, β-carotene, potassium, calcium, iron and α-linoleic acid intakes. Energy from saturated fat, cholesterol, sugar, sodium and zinc intakes decreased, while energy from total and MUFA, MUFA:SFA, fibre, vitamin E, linoleic acid and long chain omega-3 fatty acid intake increased significantly. There were no significant within-group differences for the HabDiet group between baseline and four months. At four months, the MedDiet group was consuming more total fat and MUFA, vitamin E, fibre and linoleic acid, and less SFA, sodium and zinc than the control group. While total flavonoid intake was not different between groups at four months, those in the MedDiet group were consuming more total carotenoids, and anthocyanidins were borderline significantly higher in the MedDiet group compared to the control (*p* = 0.05). Between groups, intakes of extra virgin olive oil, fruits, nuts, legumes, dairy, fish, meats and miscellaneous foods were significantly different at four months (diet*visit *p* < 0.05). Intakes of extra virgin olive oil, fruits, nuts, legumes, dairy, and fish were higher in the MedDiet group, while meat and miscellaneous food intakes were lower relative to the control group. 

Originally, 17 participants were allocated to energy levels 3 and 4, while 19 were allocated to energy level 2 and 27 to energy level 1. However, this was adjusted during the two and four week visits based on participant reports of satiety, and adherence levels. After the first month, 12 volunteers were allocated to energy level 1, 25 to energy level 2, 12 to energy level 3 and 31 to energy level 4. Mean energy intakes for energy levels 1, 2, 3 and 4 were 10 MJ, 9.6 MJ, 8.4 MJ and 7.8 MJ at four months, respectively. 

### 3.3. Serum Carotenoids, Erythrocyte Fatty Acids and 24-h Urinary Metabolites

Table 5 shows the changes within and between groups for objective markers of adherence, including urinary metabolites, carotenoids and erythrocyte fatty acids. After six months, serum β-carotene and lycopene increased significantly in the MedDiet group compared to the control group. MUFA as a percentage of total erythrocyte fatty acids increased, and SFA as a percentage of total erythrocyte fatty acids decreased. Magnesium excretion was significantly increased in the MedDiet group compared to the control at six months. There were no significant differences between groups for serum β-cryptoxanthin, α-carotene, lutein:zeaxanthin; erythrocyte content of omega-3, omega-6, omega-3:omega-6, and trans fats; or 24-h urinary excretion of sodium, potassium and calcium. For specific long chain fatty acids, there were no between group differences at 6 months, however docosahexaenoic acid (DHA) was significantly higher in the MedDiet group compared with the control at three months.

### 3.4. Exit Survey

**Figure 3:**
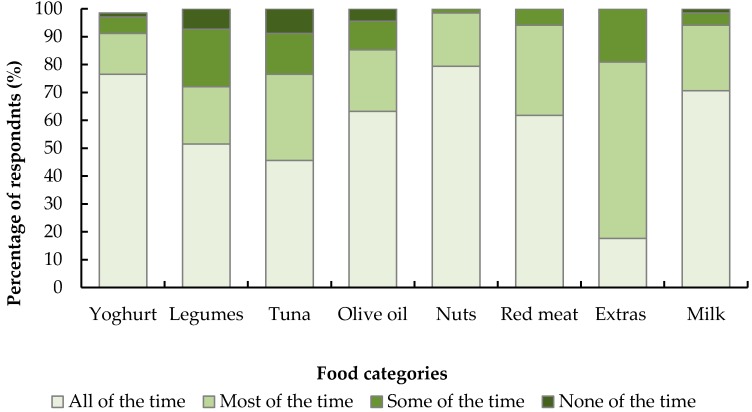
Self-reported enjoyment of requirements of the Mediterranean diet. Australian men’s and women’s (*n* = 68, age ≥ 65) answers to how well Mediterranean dietary recommendation were enjoyed/managed over a 6 month dietary intervention. Thus, an “all of the time” response indicates the dietary recommendation was enjoyed and/or well managed for the entire study duration. Yoghurt was recommended six times per week, legumes three times per week, tuna one time per week, olive oil (extra virgin) daily, nuts 4–6 times per week, red meat < 1/week, extras three serves or fewer per week and milk (skim) < 1 cup per day. Serving sizes were 170 g, 75 g, 95 g, 1 table spoon, 35 g, 100 g, 600 kJ and 250 mL for yoghurt, legumes, tuna, olive oil, nuts, red meat, extras and skim milk, respectively.

Of 70 MedDiet group participants who completed the study, 68 completed the exit survey. The responses to question 9 are shown in Figure 3. The nuts and yoghurt were very well accepted, the majority of participants enjoying these foods most or all of the time. The restriction of “extra” foods was the least accepted dietary characteristic. Approximately 75% of the group enjoyed tuna and legumes all or most of the time, and they were the least enjoyed foods of the diet. In response to question 10, more than 20% of the sample suggested eating out or with friends, holidays or special occasions were the most difficult times to continue to adhere to the diet. Other top answers included restricting particular foods, e.g., bacon, butter or beer (17%), red meat restrictions (8%), the volume of food (8%) and meeting olive oil requirements (7%). Around 5% of the participants said meeting vegetable requirements was the most difficult aspect to following the diet, while approximately 12% of the sample suggested there were no difficult aspects. Almost half the sample (47%) suggested they would aim to continue with all components, and a further 30% suggested they would continue with most components. Sixteen per cent suggested they would continue with 3 or 4 key components, with 6% suggested they would keep up 1 or 2 components. One respondent did not answer this question.

## 4. Discussion

According to a 15-point adherence score, self-reported food intakes and objective biological markers, older Australians were able to increase adherence to the MedDiet over a six-month intervention period. There was a significant increase in MUFA:SFA ratio measured from both dietary intake and erythrocyte fatty acids, and a significant increase in olive oil consumption. Serum differences in β-carotene and lycopene between groups indicated those in the MedDiet were consuming more carotenoids after four months. Carotenoid intakes and serum levels of α-carotene both increased significantly in the MedDiet group, suggesting increased intake of fruits and vegetables, which was reflected by results from the WFRs. Previous studies including the EPIC and Women’s Health Study have shown serum carotenoids to correlate with fruit and vegetable intakes [33,34,35,36]. Exit survey results indicate that, while the diet was easy to adhere to, restrictions on discretionary foods were a potential barrier, as were special occasions, holidays and eating out. Nevertheless, the vast majority of volunteers in the intervention group intended to continue most aspects of the diet after finishing the study.

The average 15-point adherence score moved from a medium to high level in the MedDiet group. In a sensitivity analysis, we also calculated the 10-point MDS, using both our sex-specific median intakes, and those from the Greek cohort of the EPIC study. All three scoring methods showed a significant increase in score amongst the MedDiet group over time compared to the control group. Scores increased by 23% according to our score, 22% according to the MDS using our medians, and 21% using the Greek EPIC medians, thus increases were similar across the three systems. Adherence was lower at all three time points when using cut-offs from the Greek data, suggesting that, although adherence did increase, the dietary intake achieved by our participants is still lower than a Mediterranean population. This could have consequences for health outcomes, however in their meta-analysis, Sofi et al. [6] showed that a two-point increase in the MDS was associated with 10% reduced risk of CVD. In the PREDIMED study adherence scores increased from approximately 8.7 to 10.6 (out of a maximum score of 14) after one year [12], which is again similar to our results, suggesting our findings are clinically relevant. The authors of PREDIMED noted their findings may not be generalizable as the population studied was Spanish, who likely had some pre-existing Mediterranean dietary practices, as well as a culturally Mediterranean setting. Similarly, in the Lyon Diet Heart Trial, where adherence to the MedDiet was high long after the intervention period had ceased, the population were French, which may be culturally more similar to traditional Mediterranean countries than modern Western nations [37]. Epidemiological studies have confirmed a higher adherence to the dietary pattern does result in reduced risk of CVD in non-Mediterranean regions [6,11]. In US populations, closer adherence to the MedDiet has been shown to reduce the risk of all-cause, cardiovascular and cancer mortality in people with pre-existing CVD [20]. Kouris-blazos et al. [18] showed in an older Melbourne cohort of Anglo-Celtic participants, that those adhering more closely to a MedDiet had reduced risk of mortality after 5 years follow-up. Based on such evidence, it is suggested the benefits of the diet are transposable [38]. It is unknown whether the increase in adherence observed in our study will result in long term dietary change, and whether this would result in improved CVD outcomes. Further study is needed in Australia to determine long term ability to adhere to the MedDiet and effects on CVD outcomes. 

In general, objective data supported the self-reported nutrient intake data, with some exceptions: changes in dietary intakes and urinary excretion were similar except for magnesium, where urinary excretion increased while dietary intake reportedly decreased, although this did not reach significance. Although 24-h urinary excretion of sodium and potassium has been linked to disease outcomes [39], many factors other than diet affect excretion of urinary metabolites on an individual level, such as balance of electrolytes, degree of hydration, race, BMI and gender [40,41]. A recent study showed correlations between dietary sodium and potassium and 24-h urinary excretion were low [41]. Although there was a significant increase in fish intake and long chain omega-3 fatty acids reported, this was not reflected by change in the total per cent omega-3 within the erythrocyte fatty acids. There was however an increase in DHA intake amongst the MedDiet group, and a between group difference at three months for DHA, suggesting an increased intake of fish compared to the control group. Serra-Majem et al. [42] found in their review that red blood cell phospholipid DHA was a robust measure of intake of DHA. Very little omega-3 fats were contributed by α-linolenic acid; almost all were contributed by fish and seafood. The predominant changes in nutrient intakes appear to be due to increased olive oil intake as the main culinary fat, and reduced intake of discretionary foods. This was reflected by a significant change in MUFA:SFA, vitamin E and SFA intakes. There was also an increased intake of total n-6 fatty acids from plant foods, which may be due to increased nut intake. This change was not reflected by change in erythrocyte fatty acid profile, where % total omega-6 was unchanged across groups and time. Although the MedDiet is typically moderate in dairy, we reported an increased intake. This could be a consequence of providing yoghurt to the participants, thereby encouraging greater consumption. In addition, baseline intake of dairy servings was below the amount recommended as part of the MedDiet. Thus, a rise in dairy intake could have been expected. Baseline dairy food intakes were similar to national averages obtained from the Australian Health Survey 2011–2012 (1.5 servings per day amongst those aged ≥2 years), which is well below national guideline recommendations [15,32]. Despite the reported increase in dairy consumption, calcium intakes did not significantly change, perhaps because of different types of dairy being consumed (e.g., white cheese instead of yellow cheese). One nutrient where a decreased intake may be of concern is zinc, which likely decreased due to decreased red meat intake. Notably however, both males and females were still meeting the estimated average requirement at four months, suggesting the diet provides adequate zinc [43]. 

Intake of Mediterranean foods including fruits, nuts, vegetables, legumes and fish increased, and non-core/non-Mediterranean foods decreased. As energy intakes did not change, core foods completely replaced the energy lost from discretionary foods. Across all four energy levels, energy intake was higher than the prescribed level, suggesting participants underestimated energy intake in their baseline WFRs. This is a common phenomenon with dietary recording [44]. In a literature review, we have previously determined that the “average” MedDiet provides a MUFA:SFA ratio of 2.1, 200 mg vitamin C, 500 μg folate, 3614 mg potassium and 31 g fibre daily—in four months participants in this study achieved a MUFA:SFA ratio of 2.2, vitamin C intake of 165 mg/day, folate intake of 487 μg/day, potassium intake of 3850 mg/day and a fibre intake of 33.5 g/day [26]. This suggests that, in most aspects, participants were able to achieve a diet nutritionally similar to a representative MedDiet by substituting with readily available Australian products. This is important when considering whether the MedDiet can be adopted in Australia. Estimations of total flavonoid intake on the MedDiet vary considerably [26]. In a recent estimation of a Mediterranean-Spanish diet which used similar calculation methods to the present study, cut-offs for the 4th and 5th quintiles were 512 and 670 mg/day, which is similar to the total flavonoid estimation we produced [45]. Interestingly only total carotenoid intakes increased significantly in the MedDiet group relative to the control, with no differences for other flavonoid subclasses. This was mainly driven by an increased intake of anthocyanidins, which are sourced primarily from red wine, although the increase in red wine intake did not reach significance when assessed based on self-reported intakes.

Exit survey results indicate that while not all components of the diet were perceived as easy to adopt, the majority of volunteers intended to continue most aspects of the diet after finishing the study. The most difficult things about following the MedDiet were the influences of friends/family, eating out and while holidaying, and food restrictions such as red meat, beer and discretionary foods. Considering the MedDiet restricts intakes of these foods below the national average intake for men and women, this is not surprising [46]. Eating out is likely to challenge due to the abundant non-core food choices available, and lack of Mediterranean options. Special occasions, such as Christmas and holidays, are culturally orientated around particular foods which might explain why participants found these occasions difficult. However, small digressions for such occasions may be acceptable if at other times the MedDiet is followed. Education is likely required to ensure appropriate choices are made when eating out, or cooking instruction may be necessary to allow persons to cook within the home in accordance with MedDiet principles. 

Limitations to this study must be acknowledged. Use of a priori adherence scores in non-Mediterranean countries has been criticised, as they do not necessarily compare intakes to a traditional MedDiet, and often exclude important food groups such as nuts [47]. We developed a novel 15-point scoring system which includes six additional food groups compared to the traditional 9-point MDS [23] to help overcome this limitation. Our score is based on baseline mean intakes, thus focuses on movement of dietary intake towards or away from a traditional MedDiet from baseline, rather than comparing intakes directly to a traditional MedDiet per se. Our sensitivity analysis revealed that although adherence increased amongst the MedDiet group, when intakes were compared to those from a MedDiet country (Greece) adherence was still medium (4.8/9) at four months. The study was amongst older, metropolitan dwelling participants, thus results cannot be extrapolated to more rural or younger populations. Self-reported dietary intakes are subject to important biases, whereby participants may knowingly or unknowingly alter or misrepresent their food and beverage intakes. Three day WFRs do not necessarily capture complete diet, for example red meat intake may have been higher than that reported if participants were consuming it on days not recorded. However, repeat measures, consecutive days and the inclusion of weekend and week days combat these limitations. The subjective markers of adherence were supported by objective markers, suggesting dietary intake data were accurate. Participants were supported by visits with a dietitian held biweekly, which is essential for providing volunteers with tools and education and has been shown to enhance compliance and outcomes in dietary trials [48]. More data are needed to assess whether a free-living population could continue following the MedDiet without trial support. Six months may not be long enough to determine whether a population can permanently adopt substantial dietary changes. Follow-up data from this trial to determine any sustained dietary changes are underway. We did not assess in detail lifestyle factors that could impact adherence such as cost of the diet, only adherence itself. The monetary cost of adhering to the MedDiet in the past has been shown to be higher than consuming a Western diet. A systematic review included two Spanish studies which both concluded that the MedDiet is more expensive than Western diets [49]. However, in a North American study, while less energy dense, the MedDiet was not associated with increased food costs. In England, one small study (*n* = 23) found that money spent on foods before and after a three-week MedDiet intervention was not significantly different [19]. In Australia, a recently published article suggested the MedDiet would be cheaper than the typical diet of Australians [50]. The investigators collected seven-day WFRs from a subsample (*n* = 20) of participants with major depressive disorders. The estimated cost of food per week from the WFRs was $138 per person, compared to an estimated cost of a modified MedDiet at $112 per week per person [50]. Thus, cost may only be a perceived barrier in Australia and education can address this concern. 

## 5. Conclusions 

In this study, a population of older, free-living Australians made substantial changes to their food and nutrient intake, resulting in changes in erythrocyte MUFA:SFA and serum carotenoids and an increased adherence to the MedDiet. From a feasibility perspective, our results suggest the MedDiet may be an option for dieticians and other health professionals to recommend to patients/clients with health issues. Importantly, data are still needed to confirm the health benefits of the MedDiet in Australia.

## Figures and Tables

**Table 1:** Baseline serum carotenoids, erythrocyte fatty acids and urinary metabolites for participants enrolled in the MedLey study, by diet group allocation.

Serum Carotenoids	MedDiet (*n* = 80)	HabDiet (*n* = 72)
β-cryptoxanthin (ng/mL)	57.5 ± 55.8	52.7 ± 42.7
Lycopene (ng/mL)	192.1 ± 133.0	191.2 ± 228.9
α-carotene (ng/mL)	92.0 ± 145.8	74.2 ± 128.7
β-carotene (ng/mL)	1287.4 ± 1489.7	1028.3 ± 1785.9
Lutein:zeaxanthin	505.2 ± 376.0	513.2 ± 238.2
Erythrocyte fatty acids	*n* = 80	*n* = 72
Total SFA (%tot)	43.3 ± 0.9	43.6 ± 1.1
Total trans fatty acids (%tot)	0.4 ± 0.1	0.4 ± 0.1
Total MUFA (%tot)	18.4 ± 0.9	18.5 ± 1.2
Total PUFA (%tot)	37.8 ± 1.1	37.2 ± 3.2
Total omega-3 fatty acids (%tot)	10.7 ± 2.6	10.4 ± 2.7
Total omega-6 fatty acids (%tot)	27.1 ± 2.7	27.1 ± 2.8
Omega-6:omega-3	2.7 ± 0.8	2.8 ± 0.8
Omega-3 index	7.4 ± 2.5	7.1 ± 2.5
Urinary metabolites	*n* = 80	*n* = 70
Sodium (mmol/24 h)	115.2 ± 51.0	119.6 ± 78.3
Potassium (mmol/24 h)	79.4 ± 24.4	78.3 ± 23.8
Calcium (mmol/24 h)	3.6 ± 1.8	4.1 ± 2.0
Magnesium (mmol/24 h)	4.6 ± 2.2	4.3 ± 1.5

Values are mean ± standard deviation (SD). MedLey, Mediterranean diet for cognitive and cardiovascular disease in the elderly study; %tot, % of total erythrocyte fatty acids; SFA, saturated fatty acids; MUFA, monounsaturated fatty acids; PUFA, polyunsaturated fatty acids; Omega-3-Index = sum of eicosapentaenoic acid and docosahexaenoic acid.

**Table 2:** Results of the sensitivity analysis to compare adherence scores using the Mediterranean Diet Score [22].

Adherence Score	Adherence Scores						*p* for Interaction
	Baseline		2 Months		4 Months		
	MedDiet	HabDiet	MedDiet	HabDiet	MedDiet	HabDiet	
15-point score	7.1 ± 1.9	7.4 ± 2.4	9.7 ± 2.4	7.7 ± 2.2	10.6 ± 1.7	7.9 ± 2.5	<0.001
10-point MDS ^1^	4.2 ± 1.7	4.2 ± 1.6	5.9 ± 1.6	4.1 ± 1.7	6.2 ± 1.4	4.4 ± 1.5	<0.001
10-point MDS ^2^	2.8 ± 1.4	2.8 ± 1.5	3.7 ± 2.1	2.6 ± 1.5	4.7 ± 1.3	3.0 ± 1.3	<0.001

Values presented are mean ± SD. *p* for dietxvisit interaction derived from linear mixed effects models to compare MedDiet and HabDiet groups across time. ^1^ Using baseline sex-specific medians. ^2^ Using baseline sex-specific medians from the Greek cohort in the EPIC study, from Trichopoulou et al. [22] MDS, Mediterranean Diet Score; MedDiet, Mediterranean diet; HabDiet, Habitual diet.

**Table 3:** Daily nutrient and flavonoid intake assessed by three-day weighed food record, for the Mediterranean and habitual diet groups.

	MedDiet (*n* = 80)			HabDiet (*n* = 70)				Between Group Difference at 4 Months ^1^	
Nutrients ^2^	Baseline	4 Months	*p* Value	Baseline	4 Months	*p* Value	Diet*Visit Interaction	Difference (95% CI)	*p* Value
Energy (kJ)	8847.2 ± 229.7	8777.8 ± 245.5	1.00	8773.9 ± 229.7	8380.1 ± 252.1	0.377	0.41	384.3 (−1070.6, 302.2)	0.27
kJ from protein (%)	19.3 ± 0.37	19.5 ± 0.4	1.00	19.3 ± 0.4	19.3 ± 0.4	1.00	0.96	0.2 (−0.9, 1.3)	0.74
kJ from fat (%)	33.5 ± 0.7	38.7 ± 0.8	<0.001	34.4 ± 0.7	35.6 ± 0.8	0.65	<0.001	3.1 (1.1, 5.2)	<0.01
kJ from saturated fat (%)	12.1 ± 0.3	9.1 ± 0.3	<0.001	12.9 ± 0.3	13.1 ± 0.3	1.00	<0.01	−4.0 (−4.9, −3.1)	<0.001
kJ from monounsaturated fat (%)	13.2 ± 0.4	19.7 ± 0.5	<0.001	13.2 ± 0.4	14.3 ± 0.5	0.24	<0.001	5.5 (4.2, 6.8)	<0.001
kJ from carbohydrate (%)	42.3 ± 0.7	37.8 ± 0.8	<0.001	41.3 ± 0.8	40.3 ± 0.8	0.77	<0.001	−2.5 (−4.7, −0.3)	0.03
kJ from alcohol (%)	4.5 ± 1.1	2.7 ± 1.2	0.40	2.9 ± 1.2	3.4 ± 0.4	1.00	0.47	−0.6 (−3.2, 2.0,)	0.67
Fat as mono (%)	42.5 ± 0.7	54.3 ± 0.7	<0.001	41.3 ± 0.7	43.1 ± 0.7	0.09	<0.001	11.2 (9.3, 13.7)	<0.001
Fat as saturated (%)	39.6 ± 0.8	25.3 ± 0.9	<0.001	41.0 ± 0.9	40.0 ± 0.9	1.00	<0.001	−14.7 (−17.1, −12.3)	<0.001
Cholesterol (mg/MJ)	33.3 ± 1.4	25.5 ± 1.5	<0.001	34.7 ± 1.5	36.1 ± 1.5	1.00	<0.001	−10.5 (−14.7, −6.3)	<0.001
Sugars (g/MJ)	12.5 ± 0.38	11.3 ± 0.36	<0.01	12.1 ± 0.4	11.6 ± 0.38	0.56	0.51	−0.3 (−1.3, 0.8)	0.62
MUFA:SFA	1.2 ± 0.1	2.2 ± 0.1	<0.001	1.1 ± 0.1	1.2 ± 0.1	0.50	<0.001	1.0 (0.9, 1.2)	<0.001
Fibre (g/MJ)	3.4 ± 0.1	3.8 ± 0.1	<0.01	3.2 ± 0.1	3.1 ± 0.1	0.80	<0.01	0.7 (0.4, 1.0)	<0.001
Vitamin C (mg/MJ)	17.6 ± 1.1	18.9 ± 0.97	1.00	15.9 ± 1.2	14.2 ± 1.0	0.53	0.21	4.7 (1.9, 7.5)	<0.01
Vitamin E (mg/MJ)	1.2 ± 0.5	1.9 ± 0.6	<0.001	1.2 ± 0.6	1.3 ± 0.6	0.74	<0.001	0.6 (0.5, 0.8)	<0.001
Total folate (µg/MJ)	54.4 ± 2.1	56.1 ± 1.2	1.00	49.8 ± 2.3	47.3 ± 2.0	1.00	0.51	8.8 (3.3, 14.4)	<0.01
Total vitamin A equivalents (µg/MJ)	130.4 ± 6.8	140.0 ± 15.4	1.00	121.3 ± 7.3	131.4 ± 16.0	1.00	0.37	8.6 (−35.5, 52.6)	0.70
B-carotene equivalents (µg/MJ)	588.7 ± 40.4	687.4 ± 53.6	0.33	473.4 ± 43.2	467.4 ± 55.6	1.00	0.31	220.0 (67.4, 372.7)	<0.01
Sodium (mg/MJ)	264.5 ± 8.8	202.3 ± 6.8	<0.001	267.7 ± 9.4	259.5 ± 7.0	1.00	<0.01	−57.2 (−76.6, −37.9)	<0.001
Potassium (mg/MJ)	447.1 ± 10.6	442.5 ± 9.0	1.00	421.6 ± 11.3	403.1 ± 9.3	0.48	0.71	39.3 (13.8, 64.9)	<0.01
Calcium (mg/MJ)	109.3 ± 4.0	105.5 ± 3.2	1.00	107.1 ± 4.3	103.9 ± 3.4	1.00	0.62	1.6 (−7.7, 10.8)	0.74
Magnesium (mg/MJ)	50.2 ± 1.3	44.2 ± 2.0	0.01	46.4 ± 1.4	41.8 ± 2.1	0.09	0.48	2.4 (−3.2, 8.1)	0.39
Iron (mg/MJ)	1.61 ± 0.4	1.57 ± 0.4	1.00	1.5 ± 0.4	1.5 ± 0.4	1.00	0.53	0.1 (−0.1, 2.1)	0.07
Zinc (mg/MJ)	1.4 ± 0.4	1.2 ± 0.5	<0.01	1.4 ± 0.4	1.5 ± 0.5	0.09	<0.001	−0.2 (−0.4, −0.1)	<0.01
Total long-chain *n*3 (mg/MJ)	50.4 ± 6.6	89.0 ± 7.1	<0.001	52.9 ± 7.0	35.9 ± 7.3	0.27	<0.001	53.1 (32.9, 73.2)	<0.001
Linoleic acid (g/MJ)	1.3 ± 0.6	1.7 ± 0.6	<0.001	1.3 ± 0.6	1.3 ± 0.6	1.00	<0.01	0.4 (0.2, 0.6)	<0.001
A-linolenic acid (g/MJ)	0.16 ± 0.01	0.18 ± 0.01	0.68	0.17 ± 0.1	0.14 ± 0.1	0.17	0.07	0.0 (0.0, 0.1)	0.03
Total flavonoids (mg/MJ)	59.7 ± 5.7	64.9 ± 5.8	0.35	68.1 ± 6.0	63.0 ± 6.0	0.04	0.94	3.4 (−16.0, 22.8)	0.73
Anthocyanidins (mg/MJ)	7.8 ± 1.0	10.0 ± 1.0	0.02	7.8 ± 1.1	6.6 ± 1.1	0.20	0.01	3.3 (0.03, 6.66)	0.05
Flavan-3-ols (mg/MJ)	45.0 ± 4.7	47.3 ± 4.8	0.51	52.6 ± 5.0	51.7 ± 5.0	0.83	0.82	−2.1 (−16.3, 20.5)	0.82
Flavanones (mg/MJ)	3.0 ± 0.4	2.3 ± 0.3	0.05	2.6 ± 0.4	1.4 ± 0.3	<0.01	0.59	0.9 (−0.1, 1.8)	0.06
Flavones (mg/MJ)	0.3 ± 0.1	0.6 ± 0.1	0.10	0.4 ± 0.1	0.3 ± 0.1	0.50	0.36	0.6 (−0.2, 1.4)	0.14
Flavonols (mg/MJ)	3.3 ± 0.3	3.6 ± 0.3	0.16	3.2 ± 0.3	3.1 ± 0.3	0.56	0.18	0.5 (−0.2, 1.3)	0.18
Isoflavones (mg/MJ)	0.1 ± 0.0	0.2 ± 0.1	0.26	0.1 ± 0.0	0.1 ± 0.1	0.82	0.48	0.2 (−0.1, 0.4)	0.25
Carotenoids (mg/MJ)	1.6 ± 0.1	2.0 ± 0.1	0.03	1.4 ± 0.1	1.3 ± 0.2	0.46	0.03	0.8 (0.5 1.2)	<0.001

Values are mean ± standard error of the mean (SEM). ^1^ Linear mixed effects models with diet*visit interaction and unstructured covariance used to determine within and between group differences, α set to 0.05. For between group differences, HabDiet values were subtracted from the MedDiet values, hence negative values indicate MedDiet group had lower intake. ^2^ Nutrient intakes expressed as either % energy or per MJ (420 kcal) to control for energy intake.

**Table 4:** Daily intake of 15 food groups assessed by three-day weighed food record for the Mediterranean and habitual diet groups.

	MedDiet (*n* = 80)			HabDiet (*n* = 70)				Between Group Difference at 4 Months	
Foods ^1^ (g/mJ/Day)	Baseline ^2^	4 Months	*p* Value	Baseline ^2^	4 Months	*p* Value	Diet*Visit Interaction	Difference (95% CI)	*p* Value
Extra virgin olive oil	0.3 ± 0.1	3.6 ± 0.2	<0.001	0.6 ± 0.1	0.8 ± 0.2	1.00	<0.001	2.9 (2.3, 3.4)	<0.001
Vegetables	18.2 ± 1.4	25.7 ± 1.6	<0.001	19.0 ± 21.8	21.9 ± 1.6	0.40	0.02	3.8 (−0.6, 8.2)	0.09
White potato	4.8 ± 0.6	3.2 ± 0.6	0.09	5.5 ± 0.7	3.9 ± 0.6	0.09	0.09	−0.7 (−2.4, 0.9)	0.37
Fruits	30.6 ± 2.0	42.4 ± 2.3	<0.001	31.1 ± 2.1	35.6 ± 2.4	0.47	0.04	6.8 (0.2, 13.4)	0.05
Nuts	1.9 ± 0.3	5.6 ± 0.7	<0.001	1.7 ± 0.3	1.9 ± 0.7	1.00	<0.01	3.8 (1.9, 5.6)	<0.001
Legumes	2.1 ± 0.5	5.2 ± 0.6	<0.001	1.7 ± 0.5	2.2 ± 0.6	1.00	<0.01	3.0 (1.3, 4.7)	<0.01
Bread	8.3 ± 0.5	9.1 ± 0.6	0.26	9.5 ± 0.6	9.0 ± 0.6	1.00	0.22	0.05 (−1.6, 1.7)	0.95
Cereals	8.2 ± 0.8	7.4 ± 0.8	1.00	7.7 ± 0.9	8.3 ± 0.8	1.00	0.40	−0.8 (−3.1, 1.4)	0.46
Dairy (all types)	30.4 ± 2.5	37.5 ± 2.2	<0.01	32.2 ± 2.7	29.8 ± 2.2	0.94	0.01	7.7 (1.6, 13.8)	0.01
Fish and seafood	4.2 ± 0.7	9.2 ± 0.7	<0.001	6.5 ± 0.7	4..8 ± 0.7	0.17	<0.001	4.5 (2.4, 6.5)	<0.001
Eggs	2.5 ± 0.4	2.2 ± 0.4	1.00	2.9 ± 0.4	3.2 ± 0.4	1.00	0.76	−1.0 (−2.1, 0.2)	0.09
Meats	8.0 ± 0.8	5.3 ± 0.8	0.03	8.8 ± 0.9	10.7 ± 0.9	<0.01	0.43	−5.4 (−7.8, −3.1)	<0.001
Red wine	8.7 ± 1.8	10.7 ± 1.5	0.62	11.9 ± 1.9	7.5 ± 1.6	0.03	0.02	3.2 (−1.2, 7.5)	0.15
Sugars	1.3 ± 0.2	1.1 ± 0.2	0.9	1.3 ± 0.2	1.2 ± 0.2	1.00	0.01	−0.1 (−0.7, 0.5)	0.70
Miscellaneous	32.1 ± 2.1	15.6 ± 1.8	<0.001	34.5 ± 2.2	23.9 ± 1.8	<0.001	0.10	−8.2 (−13.3, −3.2)	<0.01

Values are mean ± SEM. ^1^ Vegetables included all raw, cooked, canned and frozen vegetables including sweet potato. Fruits included all fresh, dried and canned fruit but excluded fruit juice/drinks. Cereals included all rice, pasta, couscous, quinoa, muesli, oats, low fat crisp breads and crackers, and wholegrain breakfast cereals. Eggs included egg dishes such as quiche. Meats included all red meat, pork and poultry. Sugars included products at least 50% sugar (e.g., honey, jam, some confectionary, table sugar). Miscellaneous included confectionary and discretionary foods, small goods, soy products, special dietary products, muesli bars, sweet bakery products and take away foods. Beverages including water, tea, coffee, soft drink, cordials, milky drinks, fruit and vegetable juices, and alcoholic drinks other than red wine were not included. ^2^ Group specific baseline means used to calculate 15-point adherence score.

**Table 5:** Changes in serum carotenoids, erythrocyte fatty acids and urinary metabolites from baseline to six months^1^.

	MedDiet Group (*n* = 82)			HabDiet Group (*n* = 70)				Between Group Difference 6 Months	
	Baseline	6 Months	*p* Value	Baseline	6 Months	*p* Value	DietxVisit Interaction	Difference (95% CI)	*p* Value
β-cryptoxanthin (ng/mL)	56.8 ± 5.6	52.7 ± 6.6	1.00	51.3 ± 5.9	49.5 ± 7.0	1.00	0.77	0.1 (−0.1, 0.2)	0.44
Lycopene (ng/mL)	184.0 ± 13.8	197.4 ± 13.4	1.00	162.1 ± 14.8	135.6 ± 13.7	1.00	<0.01	0.2 (0.1, 0.3)	<0.01
α-carotene (ng/mL)	77.6 ± 10.2	125.1 ± 15.6	<0.01	61.6 ± 10.8	55.0 ± 16.1	0.50	<0.01	0.2 (−0.0, 0.4)	0.12
β-carotene (ng/mL)	1287.4 ± 141.4	1543.8 ± 136.1	0.10	858.7 ± 150.8	851.8 ± 136.3	1.00	<0.001	0.2 (0.1, 0.3)	<0.001
Lutein:zeaxanthin	489.5 ± 30.6	524.6 ± 33.6	0.40	511.3 ± 32.2	521.8 ± 34.9	1.00	0.30	−0.0 (−0.1, −0.1)	0.65
Total erythrocyte saturated fat (%)	43.3 ± 0.1	42.8 ± 0.1	<0.001	43.5 ± 0.1	43.5 ± 0.1	1.00	<0.001	−0.7 (−1.0, −0.5)	<0.001
Total erythrocyte trans−fat (%)	0.44 ± 0.02	0.37 ± 0.02	<0.001	0.4 ± 0.02	0.4 ± 0.02	0.34	<0.001	−0.0 (−0.1, 0.0)	0.08
Total erythrocyte MUFA (%)	18.4 ± 0.1	19.3 ± 0.1	<0.001	18.5 ± 0.1	18.6 ± 0.1	1.00	<0.001	0.8 (0.4, 1.1)	<0.001
Total erythrocyte omega-3 (%)	10.8 ± 0.3	10.9 ± 0.3	1.00	10.5 ± 0.3	10.7 ± 0.3	0.30	0.24	0.2 (−0.6, 1.1)	0.62
Docosahexaenoic acid (22:6 *n*3) (%)	5.8 ± 0.14	6.1 ± 0.13	0.01	5.6 ± 0.14	5.7 ± 0.14	0.29	0.03	0.31 (−0.06, 0.68)	0.10
Eicosapentaenoic acid (20:5 *n*3) (%)	1.8 ± 0.13	1.8 ± 0.13	1.00	1.7 ± 0.14	1.8 ± 0.14	0.38	0.58	0.01 (−0.37, 0.38)	0.98
Docosapentaenoic acid (22:5 *n*3) (%)	3.0 ± 0.06	2.9 ± 0.06	<0.001	3.0 ± 0.07	3.0 ± 0.06	1.00	<0.001	−0.11 (−0.29, 0.06)	0.19
Total erythrocyte omega 6 (%)	27.1 ± 0.3	26.7 ± 0.3	0.07	27.2 ± 0.3	27.0 ± 0.3	1.00	0.50	−0.3 (−1.2, 0.5)	0.44
Omega-6:omega-3 from erythrocytes	2.7 ± 0.1	2.6 ± 0.1	0.05	2.8 ± 0.1	2.7 ± 0.1	0.15	0.12	−0.1 (−0.4, 0.1)	0.35
Omega 3 index	1.99 ± 0.3	2.0 ± 0.3	0.02	1.9 ± 0.3	2.0 ± 0.3	0.28	0.03	0.7 (−0.0, 0.2)	0.13
Sodium (mmol/24 h)	113.9 ± 5.4	107.8 ± 4.9	0.85	118.3 ± 5.7	111.5 ± 5.1	0.73	0.30	−5.5 (−20.4, 9.4)	0.47
Potassium (mmol/24 h)	79.7 ± 2.7	80.7 ± 3.0	1.00	77.9 ± 2.9	74.9 ± 3.1	1.00	0.63	5.0 (−3.2, 13.2)	0.24
Calcium (mmol/24 h)	3.6 ± 0.2	4.0 ± 0.2	0.16	4.1 ± 0.2	3.9 ± 0.2	0.99	0.13	−0.0 (−0.7, 0.6)	0.90
Magnesium (mmol/24 h)	4.4 ± 0.2	4.7 ± 0.2	0.17	4.3 ± 0.2	4.2 ± 0.2	1.00	0.12	0.6 (0.0, 1.1)	0.04

Values are mean ± SEM. ^1^ Linear mixed effects models with diet*visit interaction and unstructured covariance used to determine within and between group differences, α set to 0.05. For between group differences, HabDiet values were subtracted from the MedDiet values, hence negative values indicate MedDiet group had lower intake. Variables β-carotene, lycopene, α-carotene, β-cryptoxanthin and lutein/zeaxanthin and omega-3 index log10 transformed before analysis due to non-normal distribution. MedDiet, Mediterranean diet; HabDiet, habitual diet; MUFA, monounsaturated fatty acids.

## Supplementary Materials

The following is available online at www.mdpi.com/2072-6643/9/5/534/s1, Table S1, MedLey raw data. Table S1 presented as excel spreadsheets. Spreadsheet contains the following tabs: (1) Food intakes as grams; (2) Food intakes as grams/MJ; (3) Nutrient intakes as grams; (4) Nutrient intakes as grams/MJ; (5) Carotenoids and erythrocyte fatty acids; (6) Urinary metabolites; and (7) Flavonoid intakes. All data presented in long format. Table S2, Cut-offs used to score adherence at baseline, and two and four months for the MedLey study. Table S3, Nutrient intakes by gender and diet group at baseline and four months, compared with Australian estimated average requirements/adequate intakes. Figure S1, The MedLey study CONSORT flow diagram.
