# Supplementary material for: Older Australians Can Achieve High Adherence to the Mediterranean Diet during a 6 Month Randomised Intervention; Results from the Medley Study
Source: Nutrients. 2017 May 24;9(6):534. doi: 10.3390/nu9060534 (PMC5490513; doi:10.3390/nu9060534)
Supplement: Supplementary file 1 [file nutrients-09-00534-s001.zip › Supplementary File 2, Table S2.pdf]

nutrients

MDPI

Table S2. Cut-offs used to score adherence at baseline, 2 and 4 months for the MedLey study

15-point adherence score				10-point MDS[1]		10-point MDS[2]	
Foods (g/MJ)	MedDiet	HabDiet	Foods (g)	males	females	males	females
Extra virgin olive oil	0.3	0.6	MUFA:SFA	0.94	1.07	1.7	1.7
Vegetables	18.2	19.0	Vegetables	186.5	198.3	549.9	499.6
Potatoes	4.8	5.5	Fruits and nuts	342.3	291.3	362.5	356.3
Fruits	30.6	31.1	Legumes	0.0	0.0	9.1	6.7
Nuts	1.9	1.7	Cereals	175.3	135.2	177.7	139.7
Legumes	2.1	1.7	Fish	33.3	31.7	23.7	18.8
Bread	8.3	9.5	Meat and poultry	138	123.3	120.8	89.8
Cereals	8.2	7.7	Dairy	176.3	197.5	196.7	191.1
Dairy	30.4	32.2	Ethanol	10-50	5-25	10-50	5-25
Fish and seafood	4.2	6.5					
Eggs	2.5	2.9					
Meats	8.0	8.8					
Red wine	8.7	11.9					
Sugars	1.3	1.3					
Miscellaneous foods	32.1	34.5					

[1]Using baseline sex-specific medians.
[2]Using baseline sex-specific medians from the Greek cohort in the EPIC study, from Trichopoulou et al [1]
MedLey, Mediterranean diet for cognitive and cardiovascular health in the elderly; MDS, Mediterranean diet score; MedDiet, Mediterranean diet group; HabDiet, Habitual diet group; MUFA:SFA, monounsaturated to saturated fat ratio.

Supplementary References

1. Trichopoulou, A.; Costacou, T.; Bamia, C.; Trichopoulos, D. Adherence to a Mediterranean diet and survivial in a Greek population. *N. Engl. J. Med.* **2003**, *348*, 2599-2608. DOI: none.
