# Supplementary material for: Older Australians Can Achieve High Adherence to the Mediterranean Diet during a 6 Month Randomised Intervention; Results from the Medley Study
Source: Nutrients. 2017 May 24;9(6):534. doi: 10.3390/nu9060534 (PMC5490513; doi:10.3390/nu9060534)
Supplement: Supplementary file 1 [file nutrients-09-00534-s001.zip › Supplementary File 3, Table S3.pdf]

nutrients MDPI

Table S3. Nutrient intakes by gender and diet group at baseline and 4 months, compared with Australian estimated average requirements/adequate intakes

	MedDiet (n=80)				HabDiet (n=70)				
	Baseline		4 months		Baseline		4 months		Recommendations
Nutrients	Males	Females	Males	Females	Males	Females	Males	Females	EAR[1]
kJ from protein (%)	18.3±3.6	20.1±3.2	19.4±2.8	19.4±3.3	18.7±3.4	19.5±3.3	19.2±4.0	19.2±3.4	15-25%
kJ from fat (%)	33.6±6.6	33.6±5.3	37.2±7.7	40.0±7.0	33.6±6.9	35.1±4.5	34.9±3.9	36.2±6.7	20-35%
kJ from saturated fat (%)	12.5±2.8	11.8±2.7	8.7±1.9	9.3±1.8	12.8±3.6	12.9±2.5	12.8±2.8	13.2±3.7	<10%
kJ from monounsaturated fat (%)	13.1±4.3	13.2±2.5	19.1±4.8	20.2±4.6	12.8±3.6	13.6±3.3	13.8±2.7	14.6±4.2	NA
kJ from carbohydrate (%)	40.7±3.9	39.5±6.9	35.5±5.3	34.1±6.0	39.2±8.5	39.5±5.1	37.1±6.0	38.4±7.7	45-65%
kJ from alcohol (%)	3.4±3.4	2.7±3.2	3.4±3.8	2.3±2.2	4.6±6.1	2.2±2.8	5.0±5.0	2.3±2.8	NA
Cholesterol (mg)	337.4±158.3	288.8±182.6	221.8±88.6	224.5±92.6	278.4±131.0	316.8±122.8	297.5±117.9	295.3±142.2	NA
Fibre (g)[2]	35.4±15.5	27.4±6.5	37.6±14.6	30.9±9.7	27.9±9.6	28.2±6.9	25.1±9.4	26.2±7.2	M 30 g<br>F 25 g
Vitamin C (mg)	151.6±89.1	148.6±87.5	180.8±79.0	155.7±74.0	141.2±69.6	132.5±71.3	124.4±91.2	117.8±55.0	30 mg
Vitamin E (mg)[2]	12.7±8.4	10.0±3.5	17.8±6.2	16.8±5.4	10.2±4.4	11.0±4.3	10.3±3.4	11.3±6.0	M 10 mg<br>F 7 mg
Total folate (µg)	582.7±289.8	407.1±119.1	546.7±169.7	446.6±127.2	448.8±188.4	413.8±153.2	374.0±142.1	399.9±154.2	320 mg
Total vitamin A equivalents (µg)[2]	1107.7±522.7	1472.6±2077.0	1197.4±1055.4	1244.4±816.7	933±456.1	1163.6±615.3	1072.3±1261.3	1473.0±2497.9	M 625 µg<br>F 500 µg
β-carotene equivalents (µg)	4553.3±3106.1	5374.8±3968.6	6234.2±6410.9	6660.1±5043.8	3601.1±2590.2	4593.5±2865.6	2787.3±1940.7	4725.2±3247.7	NA
Sodium (mg)[2]	2673.7±888.1	2094.2±733.3	2095.5±763.6	1565.6±452.9	2306.7±967.8	2330.9±806.4	2214.6±633.6	2106.5±664.5	460-920 mg
Potassium (mg)[2]	4325.3±1819.8	3604.1±804.4	4199.0±1069.9	3612.0±832.1	3619.4±1032.6	3713.3±743.2	32229.2±839.9	3416.6±781.7	2800 mg
Magnesium (mg)	491.6±184.5	403.7±110.1	494.2±135.4	407.7±100.0	414.8±121.2	421.6±110.1	389.2±133.6	381.7±100.1	M 350 mg<br>F 265 mg
Calcium (mg)	990.3±418.6	920.1±336.0	1020.4±319.5	855.6±225.1	893.3±409.8	988.2±331.5	848.8±374.7	885.4±318.6	M 840 mg (>70y 1100 mg)

									F 1,100 mg
Iron (mg)	16.6±6.8	12.5±2.9	15.6±4.0	12.3±2.7	13.5±4.7	12.3±2.6	13.9±5.4	11.0±2.8	M 6 mg<br>F 5 mg
Zinc (mg)	13.9±5.0	11.4±3.4	12.1±3.5	9.9±2.4	12.3±4.2	11.5±3.3	13.1±4.2	11.4±4.6	M 12 mg<br>F 6.5 mg
Total long-chain n3 (mg)[2]	495.4±609.2	523.2±586.4	800.8±647.0	935.8±819.4	477.0±558.5	562.8±702.1	432.7±610.4	336.6±500.2	M 160 mg<br>F 90 mg
Linoleic acid (18:2n-6) (g)[2]	12.7±7.6	10.7±5.6	15.7±7.3	14.8±5.4	11.4±4.9	11.3±4.8	11.5±5.8	10.3±4.3	M 13 g<br>F 8 g
α-linolenic acid (18:5n-3) (g)[2]	1.5±0.6	1.4±0.8	1.9±2.0	1.4±0.6	1.3±0.6	1.7±0.8	1.3±0.5	1.2±0.5	M 1.3 g<br>F 0.8 g
Total flavonoids (mg)	668.0±794.9	506.8±400.2	612.0±492.0	560.4±381.6	522.9±447.1	600.0±454.1	441.2±330.3	531.5±397.0	NA
Anthocyanidins (mg)	88.3±99.8	57.2±81.8	99.9±87.3	79.2±72.2	88.0±120.5	58.8±67.6	60.2±78.5	48.8±54.8	NA
Flavan-3-ols (mg)	514.0±777.3	393.3±366.5	446.9±468.9	423.1±355.3	371.4±397.2	486.7±429.7	340.1±315.4	442.6±398.3	NA
Flavanones (mg)	28.3±32.6	22.7±25.1	22.9±23.7	20.0±26.7	26.2±29.0	19.1±24.7	17.4±23.4	9.2±15.6	NA
Flavones (mg)	3.8±13.0	6.7±33.5	9.0±26.9	5.7±12.3	10.4±45.8	5.3±18.0	0.9±0.8	3.5±11.0	NA
Flavonols (mg)	30.4±28.3	25.8±18.0	31.6±21.0	31.0±13.8	26.3±19.2	27.8±17.3	22.1±13.0	26.0±14.1	NA
Isoflavones (mg)	3.2±12.3	1.2±3.2	1.7±5.5	2.6±7.9	0.6±1.8	2.2±8.1	0.6±2.3	1.4±5.0	NA
Carotenoids (mg)	15.0±9.6	13.7±9.5	17.6±14.8	18.2±11.4	10.5±7.8	13.0±7.2	8.8±7.0	11.6±7.2	NA

Values are mean±SD

[1]Nutrient Reference Values: estimated average requirement estimates the nutrient intake per day likely to meet the needs of 50% of age and gender specific population. Estimated average requirements for older Australians (aged 51-70 and >70 years) reported. Where no estimated average requirement has been set, the adequate intake has been used. For macronutrients, the acceptable macronutrient distribution range is displayed.

[2]Adequate intake used

EAR=estimated average requirement. NA=not applicable
