# Supplementary figures and images for: Older Australians Can Achieve High Adherence to the Mediterranean Diet during a 6 Month Randomised Intervention; Results from the Medley Study
Source: Nutrients. 2017 May 24;9(6):534. doi: 10.3390/nu9060534 (PMC5490513; doi:10.3390/nu9060534)

**Figure S3. CONSORT**

**Flow Diagram**

MedLey 2013-2015

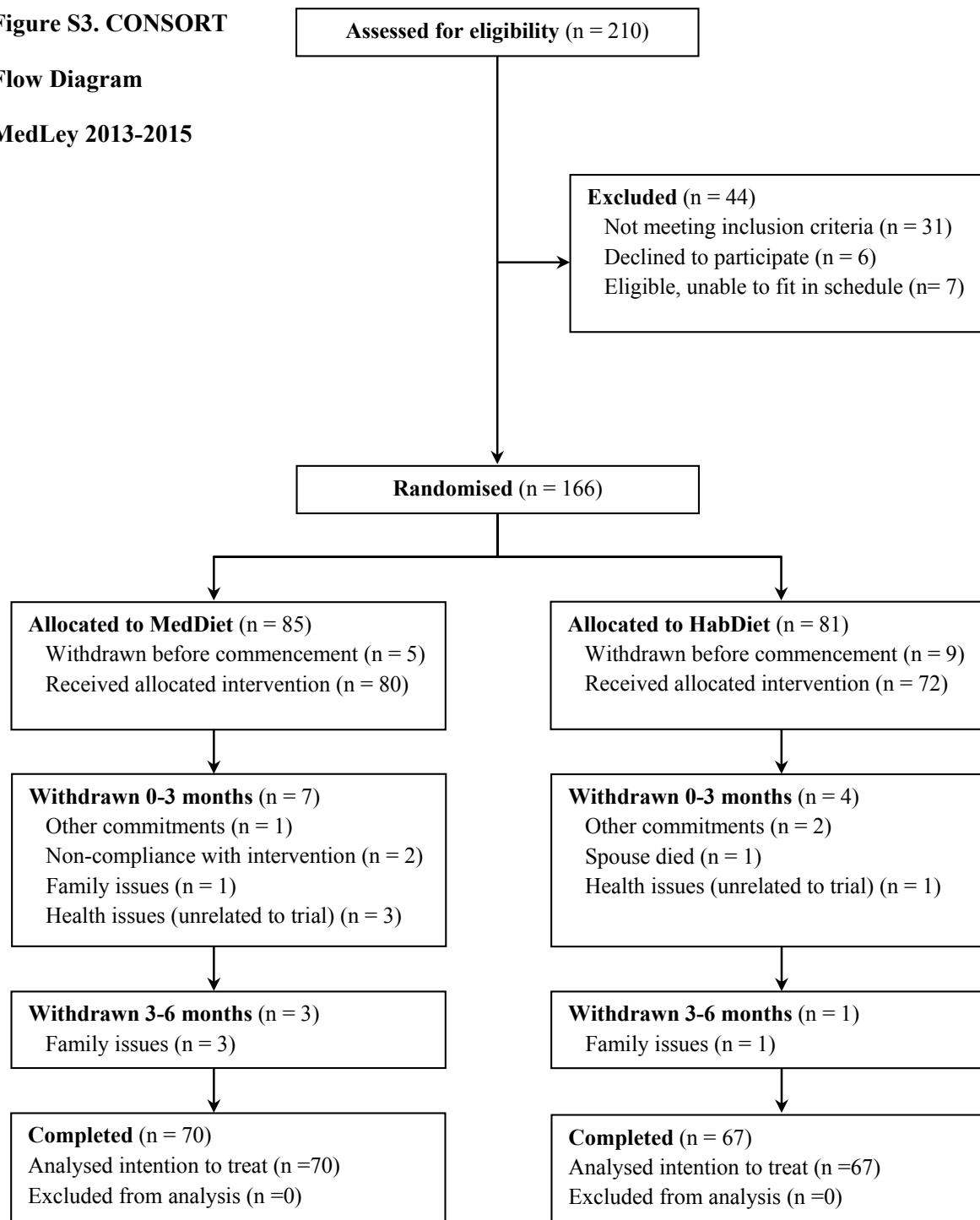

Supplement: Supplementary file 1 [file nutrients-09-00534-s001.zip › Supplementary File 4, Figure S1.pdf]
